# Effects of Anticholinergic Burden on Verbal Memory Performance in First-Episode Psychosis

**DOI:** 10.1177/07067437231179161

**Published:** 2023-05-30

**Authors:** Agnès Belkacem, Katie M. Lavigne, Carolina Makowski, Mallar Chakravarty, Ridha Joober, Ashok Malla, Jai Shah, Martin Lepage

**Affiliations:** 1 Douglas Research Centre, 5620 McGill University, Montreal, Canada; 2 Montreal Neurological Institute-Hospital, 5620 McGill University, Montreal, Canada; 3 Department of Radiology, 8784 University of California San Diego, La Jolla, CA, USA

**Keywords:** first-episode psychosis, anticholinergic burden, verbal memory

## Abstract

**Objectives:**

Antipsychotics are widely used to treat first-episode psychosis but may have an anticholinergic burden, that is, a cumulative effect of medications that block the cholinergic system. Studies suggest that a high anticholinergic burden negatively affects memory in psychosis, where cognitive deficits, particularly those in verbal memory, are a core feature of the disease. The present study sought to replicate this in a large cohort of well-characterized first-episode psychosis patients. We expected that patients in the highest anticholinergic burden group would exhibit the poorest verbal memory compared to those with low anticholinergic burden and healthy controls at baseline (3 months following admission). We further hypothesized that over time, at month 12, patients’ verbal memory performance would improve but would remain inferior to controls.

**Methods:**

Patients (*n*  =  311; low anticholinergic burden [*n*  =  241] and high anticholinergic burden [*n*  =  70], defined by a Drug Burden Index cut-off of 1) and healthy controls (*n*  =  128) completed a clinical and neurocognitive battery including parts of the Wechsler Memory Scale at months 3 and 12.

**Results:**

Cross-sectionally, using an analysis of variance, patients in the highest anticholinergic burden group had the poorest performance in verbal memory when compared to the other groups at month 3, *F*(2,430)  =  52.33, *P* < 0.001. Longitudinally, using a Generalized Estimating Equation model, the verbal memory performance of all groups improved over time. However, patients’ performance overall remained poorer than the controls.

**Conclusion:**

These findings highlight the importance of considering the anticholinergic burden when prescribing medications in the early stages of the disease.

## Résumé

**Objectifs:**

Les antipsychotiques sont largement utilisés pour traiter le premier épisode de psychose, toutefois ils peuvent avoir une charge anticholinergique, soit un effet cumulatif de médicaments qui bloque le système cholinergique. Des études suggèrent qu’une charge anticholinergique élevée peut affecter la mémoire dans la psychose, où les déficits cognitifs, en particulier ceux de la mémoire verbale, sont une caractéristique essentielle de la maladie. La présente étude cherchait à reproduire ceci au sein d'une vaste cohorte de patients avec un premier épisode de psychose. Nous avons prédit que les patients dans le groupe avec une charge anticholinergique élevée présenteraient une mémoire verbale plus réduite lorsque comparé à ceux dans le groupe avec une charge anticholinergique faible et aux contrôles en santé 3 mois après l’admission. Nous avons également supposé qu’avec le temps, au mois 12, la performance de la mémoire verbale des patients s’améliorerait, mais demeurerait inférieure à celle des témoins.

**Méthodes:**

Les patients (*n* = 311; avec une charge anticholinergique faible [*n* = 241] et avec une charge anticholinergique élevée [*n* = 70], définis par un seuil de 1 du *Drug Burden Index*) et les témoins (*n* = 128) ont été soumis à des tests cliniques et neurocognitifs (*Wechsler Memory Scale*) aux mois 3 et 12.

**Résultats:**

De manière transversale et à l’aide d’une ANOVA, les patients du groupe avec une charge anticholinergique élevée avaient une moins bonne performance à la mémoire verbale comparés aux autres groupes au mois 3 (*F*(2, 430) = 52,33, *P *< 0,001). De manière longitudinale et à l’aide d’un modèle d'Équation d'Estimation Généralisée, on observe une amélioration de la performance à la mémoire verbale dans tous les groupes au fil du temps. Cependant, la performance des patients en général reste inférieure à celle des témoins.

**Conclusion:**

Ces résultats mettent en évidence l'importance de prendre en compte la charge anticholinergique lors de la prescription de médicaments dans les premiers stades de la maladie.

## Introduction

People living with psychosis receive several medications, including antipsychotics. Developing comorbidities in psychosis is not uncommon and consequently leads to increased medication burden. Commonly used in the treatment of psychosis, anticholinergics are among the medications prescribed to patients to counter the side effects of antipsychotics.^1,3^ Evidence suggests that their substantial and cumulative effect, that is, anticholinergic burden, is associated with a significant decline in cognitive function.^4,8^

Independent of the effect of anticholinergics, cognitive impairments represent one of the core features of schizophrenia and significantly influence functional outcomes.^9,13^ Emerging evidence suggests that the anticholinergic burden from antipsychotics should be considered when prescribing medications. A high anticholinergic burden may be associated with more severe deficits in cognitive functions and, particularly, verbal memory.^14,17^

While cross-sectional studies in first-episode psychosis (FEP) suggest an association between anticholinergic burden and verbal memory performance, few studies have examined this longitudinally after initiating medication in FEP patients. Thus, a longitudinal study that includes baseline and follow-up data on cognition and medication would help shed light on this matter.

This study aimed to determine whether cognitive decline is associated with anticholinergic burden in FEP in a cross-sectional and longitudinal setting. We examined the association between anticholinergic burden and verbal memory performance between patients with a high and low anticholinergic burden (defined as a Drug Burden Index [DBI]^18^ cut-off of 1, as in previous research^14,19,21^) while covarying for factors known to affect performance (sex and age). We used cross-sectional and longitudinal data (3 and 12 months after clinical admission) to address our aims.

We hypothesized that FEP patients with higher anticholinergic burden would show poorer verbal memory performance than low anticholinergic burden and controls at month 3. We also hypothesized that both groups would show improvements in verbal memory performance over time but remain impaired relative to controls.

## Methods

### Participants

FEP patients (*n*  =  311) were recruited from the Prevention and Early Intervention Program for Psychosis (PEPP-Montreal) clinic. Entry criteria include age 18 to 35 years, no antipsychotic treatment (max. 30 days), affective or nonaffective psychotic disorder (DSM-IV), good physical health, and ability to give consent in English or French. Exclusion criteria included: minimal performance on neuropsychological tests (Intelligence Quotient [IQ] < 70), a medical history of a neurological disorder (including head injury with loss of consciousness), and a family history of neurological disorders.

For comparison purposes, nonclinical control subjects (*n*  =  128) were recruited through a centralized network of online postings. Recruitment was restricted to the same catchment area to facilitate matching controls to patients. Controls were group-matched to the FEP patients on demographic variables (sex, age and parental socioeconomic status) at recruitment. Entry criteria included age 18 to 35 years, good physical health, not taking psychotropic medications, and being able to consent in English or French. Exclusion criteria include minimal performance on neuropsychological tests (IQ < 70), history of Axis I disorders (DSM-IV) according to the Non-Patient Edition of the Structured Clinical Interview for Axis I disorders of the DSM-IV,^22^ first-order family history of psychotic disorder or hereditary neurological disease, diagnosis of substance abuse within the past 30 days, and medical history of neurological disorder (including head injury with loss of consciousness). The Douglas Research Ethics Board approved this study, and all participants provided informed consent (written and verbal).

## Setting

After admission to the PEPP-Montreal clinic program between 2003 and 2022, participants underwent a systematic assessment protocol with several checkpoints over 2 years (see study^10^ for more information). The first neurocognitive assessment took place on average 3 months after admission to the PEPP-Montreal clinic program. We considered it the baseline cognitive assessment since it was the first time cognitive performance was collected.

For this study, we only considered data at baseline (month 3; *M*  =  2.07, *SD*  =  0.89; FEP *n*  =  311, Controls *n*  =  128) and month 12 (*M*  =  12.83, *SD*  =  1.28; FEP *n*  =  107, Controls *n*  =  46) following PEPP-Montreal clinic admission once patients were clinically stabilized.

## Assessments

Patients were administered a Structured Clinical Diagnosis for DSM-IV (patient version).^22^ In months 3 and 12, structured and semistructured instruments were used: the Scale for Assessment of Positive Symptoms (SAPS) and the Scale for Assessment of Negative Symptoms (SANS),^23,24^ Duration of Untreated Psychosis (DUP), Duration of Untreated Illness (DUI) using the Circumstances of Onset and Relapse Schedule and Social and Occupational Functioning Assessment Scale (SOFAS).^25^

We also assessed medication adherence using a validated method by Cassidy et al.^26^ from a weekly adherence diary and several sources (e.g. families and case managers) at both assessments.

Neurocognitive testing was performed at both assessments using the Wechsler Memory Scale.^27^ The average of the scaled scores for Logical Memory I and II (*M*  =  10, *SD*  =  3) was used to evaluate verbal memory performance. A qualified evaluator trained by a neuropsychologist administered this neuropsychological test.

The anticholinergic burden may affect other cognitive domains. However, with the extensive literature on verbal memory, we focused on this cognitive domain to minimize the number of comparisons performed between groups for each cognitive domain.^28,29^

### Anticholinergic Burden

After carefully examining the available tools, we opted for the DBI to assess the anticholinergic burden. We consulted the systematic review of anticholinergic scales by Villalba-Moreno et al.^30^ and concluded that the DBI was the only scale that took into account the prescribed dose and the minimum effective dose, thus allowing us to quantify anticholinergic effects.^30^ Furthermore, this scale allowed us to add and combine doses of multiple medications more easily, which was essential in this sample and provided a total cumulative burden.^20^ The DBI also measures sedative medications, an important and unique feature relative to the other anticholinergic risk scales.^18,30^ The DBI was validated by a study by Hilmer et al.^18^ with a sample of 3075 patients suggesting an association between anticholinergic burden (measured with DBI) and cognitive impairments. Notably, most negative associations were found with DBI and Anticholinergic Risk Scale (ARS).^30^ Overall, this index is a useful evidence-based tool for assessing the effect of exposure to anticholinergic and sedative medications.^18,20,21,30,33^

The anticholinergic burden of medications was calculated at months 3 and 12 using the DBI from a Web Portal Software Anticholinergic Burden Calculator (https://www.anticholinergicscales.es).^19,34^ Patients were categorized into 2 groups according to a DBI score classification, that is, a DBI score <1 indicating no to low anticholinergic burden and a DBI score >1 indicating moderate to high anticholinergic burden.^18,20^ The DBI scale from the Anticholinergic Burden Calculator had only 2 classifications (labelled medium/high). We also calculated the DBI score for 3 types of DBI: total DBI, antipsychotic DBI, and other medications DBI (see Supplemental Table A for a representation of the 5 patients who showed the highest DBI scores).

### Antipsychotic Dosage

The dosage of antipsychotics was measured using Chlorpromazine Equivalent doses (CPZ-eq doses).^34^ CPZ-eq doses were calculated for the newer atypical antipsychotics,^35^ with the antipsychotic comparison chart retrieved from https://www.rxfiles.ca/rxfiles and validated by psychiatrists at the Douglas Research Center.

DBI and CPZ-eq scores were calculated for all medications taken by the patients daily, providing a cumulative score. For long-acting injectable antipsychotics, the daily dose was calculated with the total dosage divided by the number of days.^36^ For example, if an injection was given every 4 weeks, the calculation was *X* mg/ 28 days.

## Statistical Analyses

To examine differences in clinical and demographic characteristics between groups, *t*-tests and one-way analysis of variances (ANOVAs) were performed.

To examine cross-sectionally the association between anticholinergic burden and verbal memory performance at month 3, Pearson's correlations between total DBI and verbal memory performance were performed. In addition, univariate ANOVA was performed with groups as the independent variable, verbal memory performance as the dependent variable, and sex and age as covariates. We also re-ran the same ANOVA model in patients while controlling for antipsychotic dosage. For significant interactions, contrast analyses were performed.

In addition, a potential interaction between symptom severity and anticholinergic burden might exist; we therefore performed a stepwise regression using symptoms (SAPS and SANS) and anticholinergic burden (DBI) while covarying for sex and age to assess whether and by how much these predictors can explain the variability in verbal memory performance at month 3. As DBI is closely linked to medication and, therefore, symptoms, a single regression with all predictors and covariates might decrease the effect of DBI, hence the purpose of a step-by-step construction of our regression model.

A Generalized Estimating Equation (GEE) model was used with assessment (months 3 and 12) as predictors and group as outcomes to examine this association longitudinally in a subset of participants.^37^ Such a model has several advantages, including that no distributional assumption needs to be respected, supports non-normal and nested values, and not requiring a balanced set (dealing with missing values).^37^ The model was built with an independent working correlation matrix. In addition, sex and age were added as covariates. We re-ran the GEE model while controlling for antipsychotic dosage in patients and examined post hoc pairwise contrasts for significant interactions.

The CPZ-eq variable was log-transformed (ln) due to its positive skewness. All statistical analyses were performed using SPSS Inc. (version 27, released in 2020).

## Results

### Demographic/Clinical

The sociodemographic and clinical characteristics of participants in month 3 are displayed in Table 1, whereas Table 2 presents data in month 12.

**Table 1.** Clinical and Sociodemographic Characteristics of Participants at Month 3.

	FEP		Controls		FEP with longitudinal data		FEP without longitudinal data		FEP low DBI		FEP high DBI	
	(*n* = 311)		(*n* = 128)		(*n* = 107)		(*n* = 221)		(*n* = 241)		(*n* = 70)	
	Mean	*SD*	Mean	*SD*	Mean	*SD*	Mean	*SD*	Mean	*SD*	Mean	*SD*
Age (years)	23.94	4.51	24.79	4.81	24.16	4.06	24.01	4.58	23.68	4.19	22.65	4.25
Male (*n*, %)	219^a^	66.77	85	66.40	73	68.22	146^a^	66.06	165	68.22	48	68.57
Education (years)^b**^	12.15	2.51	13.96	2.13	12.50	2.43	12.32	2.52	11.98	2.33	11.24	2.71
IQ (years)^c**^	98.13	14.92	110.42	13.33	99.57	15.06	95.69	15.28	96.59	15.47	93.66	15.65
Age at onset (years)	22.34	4.97	—	—	22.36	5.13	22.30	5.04	21.99	5.01	21.96	4.29
DUP (weeks)	56.83	119.48	—	—	59.72	127.00	57.01	119.25	58.87	113.51	36.33	83.37
DUI (weeks)^d*^	330.30	308.38	—	—	353.80	315.87	330.33	307.66	308.95	284.49	320.11	312.01
SAPS	14.65	14.89	—	—	15.30	15.42	14.65	14.87	13.66	14.14	11.72	13.30
SANS	21.41	13.70	—	—	21.96	13.98	21.40	13.68	21.16	14.06	24.25	13.46
SOFAS	42.73	16.17	—	—	42.17	16.18	42.67	16.18	45.07	17.74	41.13	17.62
Adherence (%)^e*^	80.95	32.86	—	—	81.36	34.00	80.30	30.09	75.90	36.72	95.91	6.39
CPZ-eq (mg)^f**^	366.51	323.17	—	—	356.17	301.85	366.35	322.46	340.99	339.80	483.02	394.09
Total DBI	0.72	0.57	—	—	0.65	0.53	0.68	0.54	0.50	0.32	1.48	0.37
Antipsychotic DBI	0.55	0.38	—	—	0.52	0.41	0.53	0.40	0.46	0.33	0.87	0.39
Other medications DBI	0.16	0.32	—	—	0.14	0.31	0.15	0.31	0.04	0.15	0.60	0.38
Verbal memory WMS	7.50	3.15	10.79	3.06	7.86	3.20	7.79	3.22	7.70	3.15	6.92	3.03

Abbreviations: ANOVA, analysis of variance; CPZ-eq, Chlorpromazine Equivalent; DBI, Drug Burden Index (score from the anticholinergic burden calculator); DUI, Duration of Untreated Illness; DUP, Duration of Untreated Psychosis; IQ, Intelligence Quotient; SANS, Scale for the Assessment of Negative Symptoms (sum of total scores for each subscale excluding attention); SAPS, Scale for the Assessment of Positive Symptoms (sum of total scores for each subscale); *SD*, Standard Deviation; SOFAS, Social and Occupational Functioning Assessment Scale (sum of total scores); WMS, Wechsler Memory Scale 3rd and 4th Edition (average score between the scaled score Logical Memory I subtest and the scaled score Logical Memory II subtest (*M*  =  10; *SD*  =  3). ^a^Missing sex information *n*  =  3. ^b^t-tests revealed significant differences regarding years of education between patients (*M*  =  11.85*, SD*  *=*  2.44) and controls (*M*  =  14.17*, SD*  *=*  2.17), with patients having fewer years of education at month 3, *t*(350)  =  8.90, *P* < 0.001. ^c^t-tests revealed significant differences regarding IQ between patients (*M*  =  95.90*, SD*  *=*  15.38) and controls (*M*  =  108.56*, SD*  *=*  13.29), with patients having a lower IQ at month 3, *t*(453)  =  8.18, *P* < 0.001.^d^ t-tests reported differences regarding DUI between patients who remained in the study (*M*  =  362.17*, SD*  *=*  297.87, *n*  =  104) compared to patients who did not (*M*  =  286.28*, SD*  *=*  283.57, *n* =  202), where patients with follow-up data had longer DUI, *t*(304)  =  2.17, *P* < 0.05. ^e^One-way ANOVA revealed differences between patient groups, where patients with high DBI had higher adherence, *F*(1,60)  =  4.07, *P* < 0.05, compared to patients with low DBI at month 3. ^f^One-way ANOVA revealed differences between patient groups, where patients with high DBI had higher antipsychotic dosage, *F*(1,263)  =  8.25, *P* < 0.05, compared to patients with low DBI at month 3. *P < 0.05, **P < 0.001.

**Table 2.** Clinical and Sociodemographic Characteristics of Participants at Month 12.

	FEP with longitudinal data		Controls		FEP low DBI		FEP high DBI	
	(*n* = 107)		(*n* = 46)		(*n* = 48^a^)		(*n* = 16^a^)	
	Mean	*SD*	Mean	*SD*	Mean	*SD*	Mean	*SD*
Age (years)	25.22	4.17	26.42	4.40	24.98	4.03	23.77	4.03
Male (*n*, %)	73	68.22	30	65.00	16^b^	33.33^b^	8^c^	50.00^c^
Education (years)	12.98	2.65	14.11	4.40	12.30	2.64	10.73	3.15
SAPS	5.38	7.85	—	—	4.26	5.93	8.14	11.20
SANS	13.62	15.19	—	—	14.33	17.01	13.71	12.52
SOFAS	62.44	18.04	—	—	62.86	16.41	60.38	16.45
Adherence (%)	84.82	33.00	—	—	77.44	41.22	94.38	8.95
CPZ-eq (mg)	269.92	212.70	—	—	282.20	247.63	293.30	233.85
Total DBI	0.68	0.63	—	—	0.61	0.62	1.15	0.86
Antipsychotic DBI	0.49	0.36	—	—	0.46	0.37	0.70	0.38
Other medications DBI	0.27	0.52	—	—	0.15	0.41	0.45	0.63
Verbal memory WMS	9.94	3.49	11.89	2.68	9.17	3.32	8.66	3.78

Abbreviations: CPZ-eq,  Chlorpromazine Equivalent; DBI, Drug Burden Index (score from the anticholinergic burden calculator); DUI, Duration of Untreated Illness; DUP, Duration of Untreated Psychosis; SANS, Scale for the Assessment of Negative Symptoms (sum of total scores for each subscale excluding attention); SAPS, Scale for the Assessment of Positive Symptoms (sum of total scores for each subscale); SD, Standard Deviation; SOFAS, Social and Occupational Functioning Assessment Scale (sum of total scores); WMS, Wechsler Memory Scale 3rd and 4th Edition (average score between the scaled score Logical Memory I subtest and the scaled score Logical Memory II subtest (*M* = 10; *SD* = 3). ^a^Missing medication information *n* = 60. ^b^Missing sex information *n* = 24. ^c^Missing sex information *n* = 2.

### Cross-Sectional Association of Anticholinergic Burden

Pearson's correlations at month 3 revealed significant negative correlations between verbal memory and CPZ-eq (*r*  =  −0.174, *P* < 0.01), as well as verbal memory and total DBI (*r*  =  −0.143, *P* < 0.05). Pearson's correlations at month 3 revealed positive and significant correlations between CPZ-eq and total DBI (*r*  =  0.386, *P* < 0.01). A correlation at baseline between verbal memory performance and DBI was calculated. The observed negative associations were for total DBI (*r*  *=*  −0.146, *P*  =  0.010) and antipsychotic DBI (*r*  =  −0.141, *P*  =  0.013), indicating a significant effect of antipsychotics on the observed anticholinergic effects.

**Figure 1.**
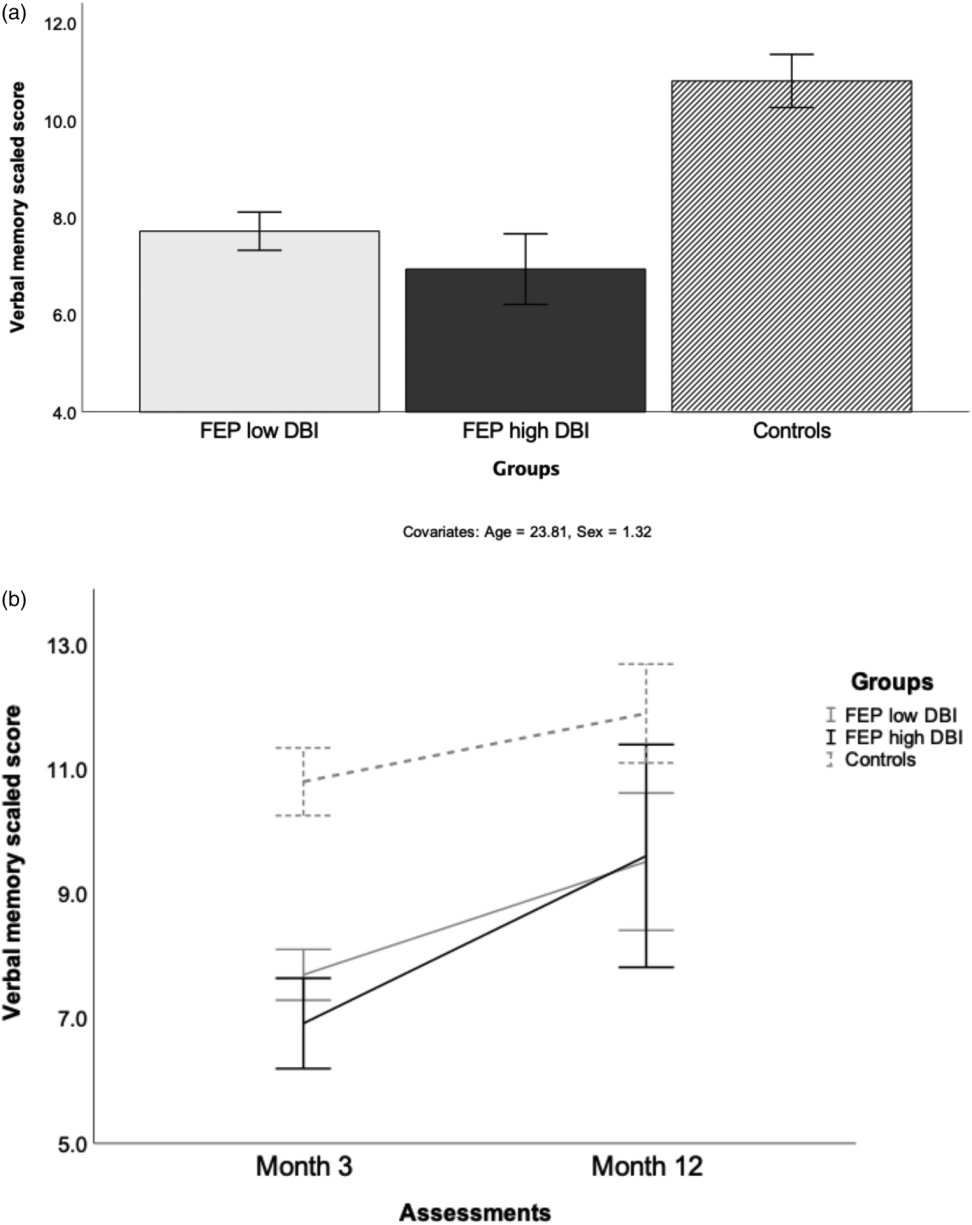
Cross-sectional association and longitudinal effects of anticholinergic burden on verbal memory performance between groups at months 3 and 12: (A) month 3 (*P* < 0.001), (B) month 12 (*P* < 0.001).

Abbreviations: DBI, Drug Burden Index; FEP, First-Episode Psychosis.

Univariate ANOVA revealed a cross-sectional association between anticholinergic burden and verbal memory performance between groups, *F*(2, 430)  =  52.33, *P* < 0.001 (Figure 1A).

Pairwise between-group comparisons revealed poorer verbal memory performance in patients with low DBI relative to controls at month 3 (Δ = 3.11, 95% CI, 2.46 to 3.76, *P* < 0.001). Pairwise between-group comparisons revealed poorer verbal memory performance in patients with high DBI compared to patients with low DBI at month 3 (Δ = 0.79, 95% CI, 0.18 to 1.56, *P* < 0.05).

As an additional analysis, using the same ANOVA model, the results indicated that after controlling for antipsychotic dosage in patients only, this effect was not significant, *F*(1, 260)  =  1.55, *P*  =  0.214.

Regarding a possible interaction between symptoms and anticholinergic burden, our results suggest that for every 1-unit increase in SAPS, verbal memory performance will decrease by 0.03 (*β* = −0.03, *P* < 0.05), and for every 1-unit increase in SANS, verbal memory performance will decrease by 0.05 (*β* = −0.05, *P* < 0.001; *R*^2^  =  0.11, *F*(4,314)  =  9.24, *P* < 0.001). Regarding anticholinergic burden, our results suggest that for every 1-unit increase in DBI, verbal memory performance will decrease by 0.75 (*β* = −0.75, *P* < 0.05; *R*^2^  =  0.07, *F*(3,306)  =  7.36, *P* < 0.001).

### Longitudinal Effects of Anticholinergic Burden

GEE analysis revealed a significant main effect of group (*χ*^2^  =  51.26, df  =  2, *P* < 0.001), a significant main effect of time (*χ*^2^  =  25.70, df  =  1, *P* < 0.001) and a marginally significant interaction between group and time (*χ*^2^  =  5.92, df  =  2, *P*  =  0.052; Figure 1B).

Pairwise comparisons between groups revealed no significant mean difference between patients with high DBI and low DBI at month 12 (Δ = 1.07, 95% CI, −1.07 to 3.22, *P*  =  0.322). Pairwise within-group comparisons revealed improved verbal memory performance in month 12 compared to month 3 for controls (Δ = 1.04, 95% CI, 0.30 to 1.78, *P* < 0.05), for patients with low DBI (Δ = 2.60, 95% CI, 1.49 to 3.70, *P* < 0.001), and patients with high DBI (Δ = 2.31, 95% CI, 0.37 to 2.26, *P* < 0.05).

As an additional analysis, using the same ANOVA model, the results indicated that after controlling for antipsychotic dosage, it remained only significant for time (*χ*^2^  =  18.06, df  =  1, *P* < 0.001).

## Discussion

Our findings suggest that patients with the highest anticholinergic burden group had the poorest verbal memory performance compared with those in the low anticholinergic burden group at month 3. This finding agrees with several cross-sectional studies investigating the association between anticholinergic burden and verbal memory.^5,7,14,17,28,38^ However, this association is not maintained after controlling for antipsychotic dosage.

Many of our FEP patients took multiple medications daily that could have a significant cumulative anticholinergic burden.^39,40^ Approximately 132 of our patients received the same primary antipsychotic at months 3 and 12 with the same route of administration, though with some changes in dosage. However, it is possible that in our sample, at an early stage of the disease, the most used medications are antipsychotics and at higher doses than other medications, which is a limitation, and it remains challenging to dissociate from medication effects on verbal memory. Improvement in symptoms could also explain the increase in cognitive performance over time. Other factors have also been shown to influence verbal memory performance (e.g. age of onset, sex, and severity of negative symptoms). However, these did not differ significantly between our DBI patient groups.^41,44^

Evidence suggests that people with schizophrenia have reduced central cholinergic activity and reduced muscarinic receptor expression;^45,46^ thus, taking multiple medications with anticholinergic burden could saturate muscarinic receptors, especially the M1 receptor, which are essential for cognitive and verbal learning.^46,48^ Moreover, developing tolerance to an anticholinergic medication may occur in patients and increase binding to muscarinic receptors in the brain, which could have contributed to our results. In addition, severely ill patients are more likely to receive higher doses of medication.^49^ This detail may also explain why, by adding antipsychotic dosage as a covariate, the initial results disappear. However, controlling for antipsychotic dosage could also remove important information about the anticholinergic burden since both are calculated from the same dose of medication.^18,35,50^

Differences between cross-sectional studies may be due to the use of different measures of anticholinergic burden.^30,51,52^ Many studies examined the anticholinergic burden only by using a scale that does not consider the daily dose and does not examine the cumulative burden of all medications patients take. Our more comprehensive calculation of cumulative burden and our consideration of all medications’ daily doses could explain the differences observed.

Our results suggest increased verbal memory performance in both controls and patients, which may indicate a practice effect over time. At month 12, no significant difference was observed between high and low anticholinergic burden on verbal memory performance. These results replicate previous longitudinal studies that also failed to find any effect of anticholinergic burden over time, including Ballesteros et al.,^14^ who did not detect a significant effect of an anticholinergic burden on verbal memory at a 2-year follow-up, and Tracy et al.,^53^ who reported no significant effect of serum anticholinergic levels on verbal memory after 1 week. However, some longitudinal studies have found that tapering high anticholinergic burden medications (e.g. biperiden) positively impacted cognition, with improved verbal memory.^2,3,54,55^

As with cross-sectional studies, results from longitudinal studies are inconsistent; several reasons may account for this. Our sample, with an average age of 23.94 years, is younger than some longitudinal studies, such as the study by Tracy et al.,^53^ with an average age of 43.86 years. A younger sample may have increased central cholinergic activity and muscarinic receptor expression, resisting long-term consequences of anticholinergic burden.^46,56,57^ Furthermore, a decrease in the density of receptors in the cholinergic system is more prevalent in severe and chronic schizophrenia than in the early stages of the disease.^58^ We also believe that polypharmacy (due to the increased medication burden with aging and changes in prescribing practices) may be an essential factor to consider in future studies, given the increased use of anticholinergics in recent years.^59,60^

A few limitations in this study can be identified. The sample size was small at month 12, making it challenging to maintain a representative sample in each group of participants over time. In addition, our first neurocognitive assessment took place 3 months after admission to the program and was considered baseline cognitive performance. However, we recognize this as a potential limitation of our study. Studying the effects of medication on cognition remains challenging as it is difficult to discriminate whether the changes are due to the severity of the illness or to the medication itself.^61^ In addition, patients with substance dependence (as defined in DSM-IV) were excluded, but those with substance abuse were included. This may be a significant limitation, as an interaction between the substance used and the medications taken as part of the treatment may occur.^62,63^ Furthermore, despite the many advantages of using the online calculator, it still has some limitations compared to other risk scales. For example, some medications, such as lurasidone, were not included in the calculator.^19^ Our results may therefore differ if another anticholinergic burden scale was used. In addition, medications that can be taken as needed have yet to be included in the DBI and CPZ-eq calculations.

The classification of the DBI at both assessments can also be considered a limitation. For example, a hypothetical patient could have a DBI of 0.98 (low DBI) at month 3 and a DBI of 1.02 (high DBI) at month 12 due to an increase in the same medication dosage, leading to a somewhat arbitrary group definition. The study of medication dosage also has several limitations due to the variability of each individual and the pharmacological profiles of antipsychotic medications, resulting in the problematic calibration of dose equivalents.

We also recognize that adopting a binary classification of DBI raises some limits to our study. We relied on the classification (medium/high) from the Anticholinergic Burden Calculator and the validated study by Hilmer et al.^18^ We also performed statistical analyses with 3 groups of DBI, that is, low (0 to 1), medium (1 to 2), and high (>2) risk, but we did not have a sufficient sample in the latter group (*n*  =  8). However, adopting a categorical approach as opposed to a continuous measure of anticholinergic also has statistical advantages where the distribution of values is manageable, and we are not assuming linearity between our variables. In addition, as our patients were between 18 and 35 years old and in the early stages of the disease, we expect that there will be more medium-risk patients than high-risk, as many of them have just started treatment (max. 30 days), often at minimal therapeutic doses.

In summary, our cross-sectional results suggest that FEP patients in the highest anticholinergic burden group had the poorest verbal memory performance compared to those in the low anticholinergic burden group and healthy controls. Longitudinal findings indicate that although verbal memory performance in all groups improved over time, all FEP patient groups had poorer verbal memory performance than controls. However, the addition of antipsychotic dosage as a covariate removed these effects. Despite the effectiveness of antipsychotics in relieving psychotic symptoms, it appears that their anticholinergic properties, as well as those of other medications taken by FEP patients, may have a detrimental effect on cognitive performance. Studies with a more extended follow-up period that consider the occupancy of dopaminergic receptors and that consider adherence in the calculation of the DBI may be helpful to understand better how medication in FEP affects cognition. Given that cognitive deficits appear in the early stages of psychosis, our results highlight the need to consider the anticholinergic burden when prescribing medications.

## Supplemental Material

sj-docx-1-cpa-10.1177_07067437231179161 - Supplemental material for Effects of Anticholinergic Burden on Verbal Memory Performance in First-Episode Psychosis Click here for additional data file. Supplemental material, sj-docx-1-cpa-10.1177_07067437231179161 for Effects of Anticholinergic Burden on Verbal Memory Performance in First-Episode Psychosis by Agnès Belkacem, Katie M. Lavigne, Carolina Makowski, Mallar Chakravarty, Ridha Joober, Ashok Malla, Jai Shah and Martin Lepage in The Canadian Journal of Psychiatry
