# Supplementary material for: Effects of Anticholinergic Burden on Verbal Memory Performance in First-Episode Psychosis
Source: Can J Psychiatry. 2023 May 30;68(12):894–903. doi: 10.1177/07067437231179161 (PMC10657580; doi:10.1177/07067437231179161)
Supplement: sj-docx-1-cpa-10.1177_07067437231179161 - Supplemental material for Effects of Anticholinergic Burden on Verbal Memory Performance in First-Episode Psychosis [file sj-docx-1-cpa-10.1177_07067437231179161.docx]

**Table A.** Drug Regimen of five FEP patients with the Highest DBI Scores

| Patients  (*n*=5) | Medication  Name | Daily Dose (mg) | Total CPZ-eq  (mg/day) | Total DBI | AP  DBI | Other DBI |
| --- | --- | --- | --- | --- | --- | --- |
| A | Quetiapine  Lamotrigine  Pregabalin | 400.00  200.00  425.00 | 532.00 | 2.52 | 0.89 | 1.63 |
| B | Aripiprazole^a^  Lurasidone  Fluoxetine  Lorazepam  Clonazepam  Pregabalin | 19.05  100.00  30.00  1.00  0.12  375.00 | 253.90 | 2.55 | 0.66 | 1.89 |
| C | Quetiapine  Citalopram  Benzatropine | 600.00  40.00  50.00 | 1 002.00 | 2.71 | 0.92 | 1.79 |
| D | Olanzapine  Haloperidol  Benzatropine  Diphenhydramine | 35.00  15.00  2.00  25.00 | 2 104.05 | 2.92 | 1.79 | 1.13 |
| E | Quetiapine  Paliperidone^a^  Fluoxetine  Zopiclone  Lithium  Lamotrigine | 50.00  3.56  20.00  7.50  900.00  100.00 | 245.07 | 3.03 | 1.04 | 1.99 |

Abbreviations: AP = Antipsychotic, CPZ-eq = Chlorpromazine Equivalent Doses, DBI = Drug Burden Index, Other = Other medication; ^a^ Long-acting injection.
